# Stable clocks, rewired rhythms: circadian remodeling in skeletal muscle during aging, cachexia, and type 2 diabetes

**DOI:** 10.1093/lifemeta/loag021

**Published:** 2026-07-15

**Authors:** Francielly Morena, Mark R Viggars, Natalie Bohmke, Theodore E Ewachiw, Karyn A Esser

**Affiliations:** Department of Physiology and Aging, University of Florida, Gainesville, FL 32611, United States; Department of Physiology and Aging, University of Florida, Gainesville, FL 32611, United States; Department of Physiology and Aging, University of Florida, Gainesville, FL 32611, United States; Department of Physiology and Aging, University of Florida, Gainesville, FL 32611, United States; Department of Physiology and Aging, University of Florida, Gainesville, FL 32611, United States

**Keywords:** muscle, rhythmic gene expression, circadian, atrophy, sarcopenia

## Abstract

In mammals, nearly every cell contains an intrinsic circadian clock that functions both as a timekeeping system and an environmental sensor, integrating external cues to maintain alignment between internal physiology and the external environment. While the core clock machinery is broadly conserved across tissues, its downstream rhythmic gene programs are highly tissue-specific and essential for maintaining cellular and physiological homeostasis. In the skeletal muscle, rhythmic program dysregulation has emerged as a common denominator in many unfavorable conditions. However, high-resolution circadian time-course studies in the muscle remain limited. In this review, we examine current evidence on the behavior of the skeletal muscle molecular clock and rhythmic transcriptional programs across aging, cancer-induced muscle atrophy (cachexia), and type 2 diabetes (T2D). Despite distinct pathological contexts, all three conditions undergo substantial condition-specific remodeling of the muscle rhythmic gene program, often converging on biological processes such as lipid metabolism and chromatin regulation. Collectively, available data suggest that circadian dysfunction in these conditions arises not from collapse of the core molecular oscillator but from progressive rewiring of rhythmic transcriptional programs despite relative preservation of core clock integrity. We discuss emerging mechanisms—including metabolic remodeling, glucocorticoid signaling, chromatin regulation, and noncanonical clock regulators—that may underlie this process. Moving forward, multi-omics studies integrating transcriptomic, proteomic, metabolomic, and epigenomic time-series analyses will be essential to distinguish mechanisms responsible for condition-specific rhythmic gene regulation. A clearer understanding of how rhythmic gene programs are rewired may reveal new opportunities to restore temporal coordination and improve skeletal muscle health across diverse pathological conditions.

## Introduction

The scientific study of circadian rhythms dates back to 1729, when Jean-Jacques d’Ortous de Mairan observed that the mimosa plant continued its daily leaf movements (nyctinasty) even in constant darkness, suggesting the presence of an internal clock [1]. Nearly 250 years later, Seymour Benzer and Ronald Konopka provided the first genetic evidence for such an internal timing mechanism by identifying fruit fly mutants with abnormal circadian behaviors [2]. Their work identified the gene *Period* (*Per*), a discovery that initiated decades of research uncovering a highly conserved and robust molecular system that regulates circadian rhythms across species, from cyanobacteria to humans [3, 4].

At the cellular level, the mammalian molecular clock operates through a transcriptional–translational feedback loop composed of more than a dozen transcriptional regulators. On the positive arm of this loop, the heterodimer brain and muscle ARNT-like 1:circadian locomotor output cycles kaput (BMAL1:CLOCK) (or neuronal PAS domain protein 2 [NPAS2] in the absence of CLOCK in the skeletal muscle [5, 6]) acts as a transcriptional activator of E-box–containing genes, including its own negative regulators, *Period* (*Per1/2*) and *Cryptochrome* (*Cry1/2*) [7, 8]. Following translation, PER:CRY protein stability is tightly regulated by conserved kinases and E3 ligases (e.g., casein kinase 1 epsilon/delta [CK1ε/δ], F-box and leucine-rich repeat protein 21 [FBXL21]) in both the cytoplasm and the nucleus [9]. During the early inactive phase of mammals, PER and CRY proteins accumulate in the cytoplasm, often as a complex. This accumulation facilitates binding of karyopherin subunit beta-1 (KPNB1) to PER, promoting nuclear translocation of the PER:CRY complex and subsequent repression of BMAL1:CLOCK activity [10, 11]. Beyond the core loop, auxiliary clock proteins such as nuclear receptor subfamily 1 group D member 1 (NR1D1, also known as REV-ERBα), NR1D2 (also known as REV-ERBβ), retinoic acid-related orphan receptor alpha (RORA), and retinoic acid-related orphan receptor C (RORC) further modulate clock function by repressing or activating *Bmal1* transcription. The timing of the transit and interactions among clock components is governed by coordinated processes of transcription, translation, post-translational modification, and protein degradation. Together, these mechanisms generate about 24-h oscillations that provide each cell with an intrinsic timekeeping system.

Beyond essential timekeeping, the BMAL1:CLOCK heterodimer binds to thousands of sites across the genome and modulates a program of daily gene expression, known as clock output. While the core clock mechanism is ubiquitous across all tissue types, the clock output is largely tissue-specific. The tissue specificity is associated with interactions between clock factors and lineage-specific transcription factors, such as the hepatocyte nuclear factor 4A (HNF4A) in the liver [12] and the myogenic differentiation 1 (MYOD1) [13] in the muscle, eliciting both epigenetic and transcriptional mechanisms supporting tissue-specific programs. Tissue-specific programs are essential for cellular homeostasis and tissue-specific physiological functions. It is important to note that not all rhythmically expressed genes (REGs) are direct outputs of the core clock mechanism. Several groups have used genetic mouse models to demonstrate that rhythmic gene expression can also result from clock-independent mechanisms, including environmental, rhythmic systemic, or behavioral cues such as neuroendocrine rhythms or feeding behavior [14]. These genes, often referred to as REGs, may therefore exhibit circadian oscillations without being mechanistically coupled to the mole­cular clock. Therefore, within this review, we will utilize rhythmic gene programs as an umbrella term that encompasses both clock-dependent and clock-independent rhythmic genes.

In mammals, virtually every nucleus harbors its own molecular clock. The clock mechanisms are intrinsic timers that also act as environmental sensors, integrating diverse cues (e.g., light, temperature, feeding, and exercise) to support coherence between internal rhythms and the external environment. Importantly, circadian health depends not only on synchrony between internal clocks and external cues but also on coordination among clocks within the body. The suprachiasmatic nucleus (SCN) of the hypothalamus serves as the central pacemaker, entrained by daily light–dark cycles and orchestrating neurohumoral signals that promote temporal alignment across peripheral tissue clocks. Nevertheless, peripheral clocks retain a degree of autonomy and can respond to non-photic, SCN-independent zeitgebers. For example, feeding schedules and glucocorticoid signaling have been shown to influence peripheral clock phase independently of direct SCN input, particularly in tissues such as the liver and the lung, highlighting the importance of behavioral and hormonal cues in peripheral clock entrainment [15, 16]. More recent studies and reviews further highlight the importance of behavioral and metabolic cues, including feeding-fasting cycles, in the synchronization of peripheral clocks [17, 18].

Although beyond the primary scope of this review, physiological interventions such as time-restricted feeding [14, 15, 18] and scheduled exercise [19] have been shown to influence the phase of peripheral tissue clocks. In general, these behaviors elicit system-wide cues that are thought to contribute to the coordination of tissue-specific transcriptional programs and physiological processes with environmental and behavioral cycles. In contrast, chronic misalignment of clocks between peripheral tissues and/or the central clock has been associated with pathophysiology across multiple organ systems. For example, social and work schedules (e.g., shift work), lifestyle behaviors (e.g., night eating), or disease states can drive misalignment of tissue circadian clocks [20]. These insights highlight the importance of studying circadian regulation not only within individual tissues but also between tissues, such as the skeletal muscle and the liver, where crosstalk between clock-controlled processes is intimately tied to metabolic regulation and disease progression [21].

Despite this importance, circadian time-course studies remain modest in number even at an individual tissue resolution, largely due to the logistical and financial challenges of longitudinal tissue collection and the downstream assays required to assess not only core clock function but also transcriptional, metabolic, and phy­siological outputs. This limitation is particularly concerning in the context of musculoskeletal health. According to the World Health Organization, approximately 1.7 billion people live with musculoskeletal conditions, such as sarcopenia, an age-associated, involuntary loss of skeletal muscle function and mass, which collectively represent the leading cause of years lived with disability (YLD) worldwide (17% of all YLDs) [22]. In this review, we synthesize current findings and explore (i) the workings of the skeletal muscle molecular clock in pathological states; (ii) the condition-specific rewiring of rhythmic gene programs and their biological implications; and (iii) potential mechanisms through which condition-specific rhythmic landscapes are modulated. By distinguishing preservation of the core oscillator from the desta­bilization of rhythmic outputs, we aim to clarify how circadian systems contribute to muscle health and maladaptation, and thus identify potential therapeutic opportunities.

## Core clock integrity versus rhythmic gene program instability in disease

### The behavior of the molecular clock mechanism: stable positive arm and vulnerable negative arm

**Figure 1:**
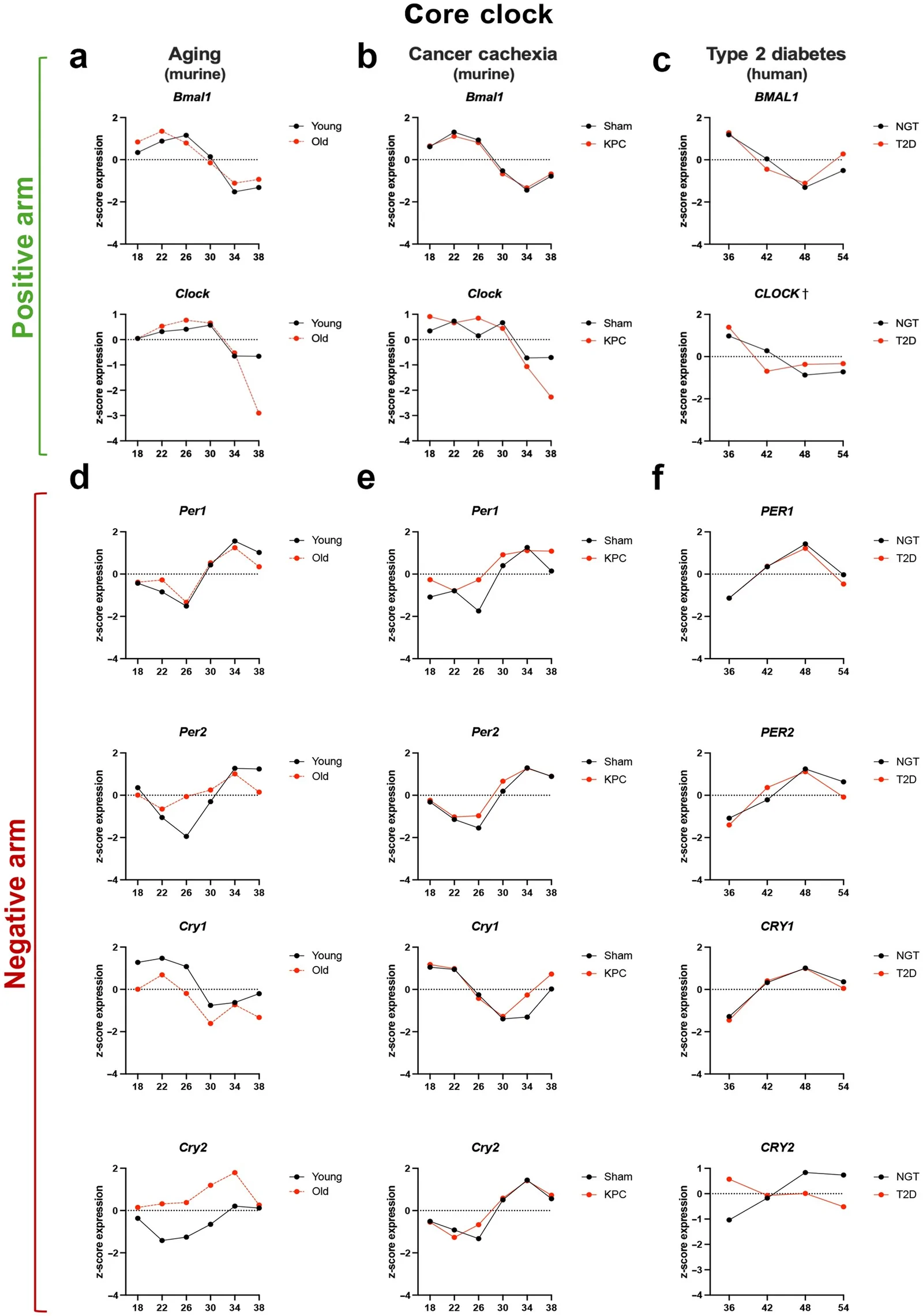
Core clock gene expression across skeletal muscle–affecting conditions. Circadian time-course expression of core clock genes in skeletal muscle under conditions of aging, cancer cachexia, and type 2 diabetes is shown. (a–c) Positive arm clock genes. (d–f) Negative arm clock genes. Gene expression is shown as Z-scored counts and plotted across a 24-h circadian cycle. ^†^Reported as exhibiting a statistically significant change (loss) of rhythmicity in the original publication. No additional statistical analyses were performed in this review.

**Figure 2:**
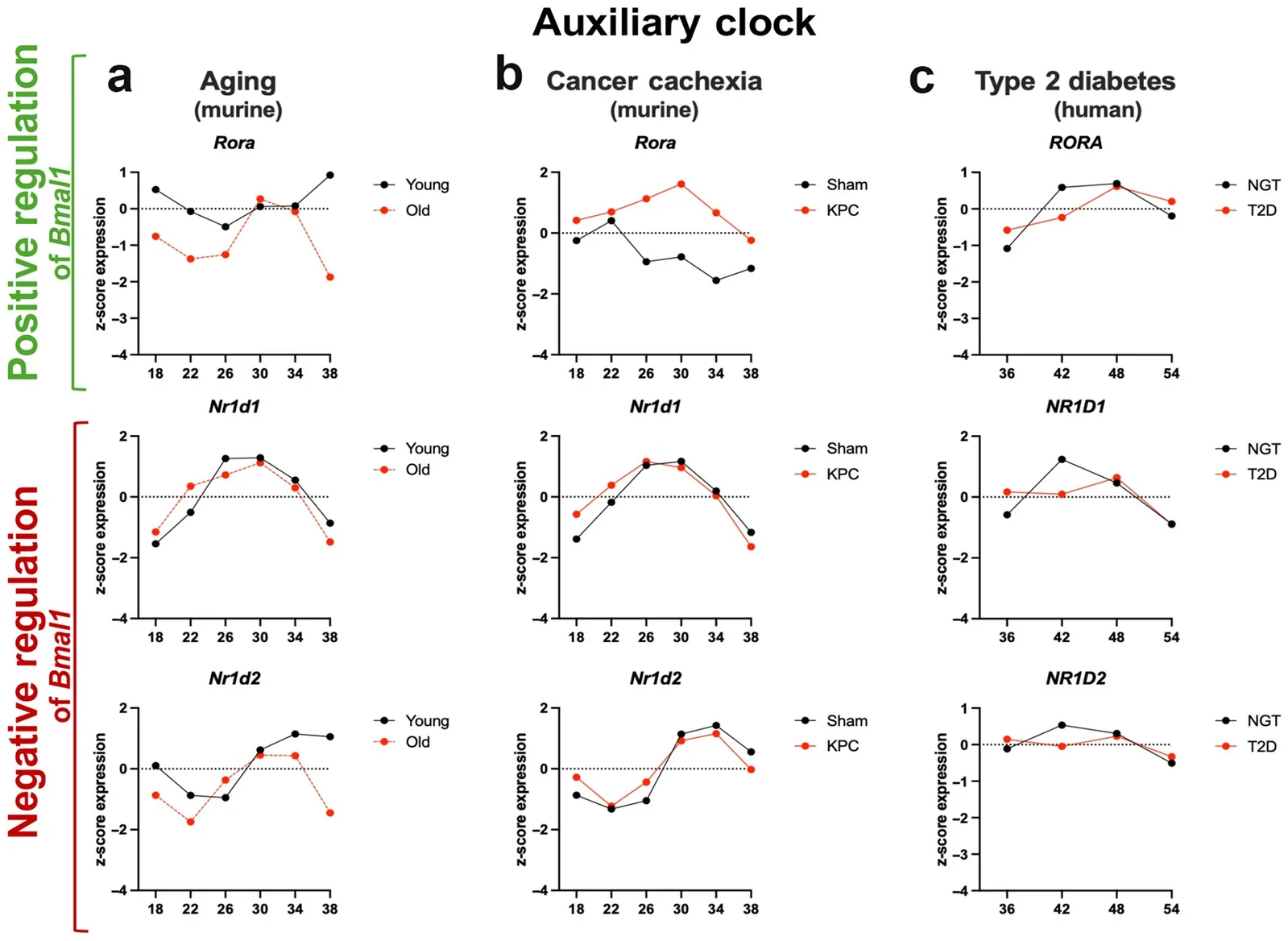
Auxiliary clock gene expression across skeletal muscle–affecting conditions. (a–c) Circadian time-course expression of auxiliary clock genes in skeletal muscle during aging (a), cancer cachexia (b), and type 2 diabetes (c). Gene expression is represented as Z-scored counts and plotted over a 24-h circadian cycle.

The study of circadian behavior, molecular clocks, and rhythmic gene programs requires appropriately frequent sampling—typically every 2–6 h for gene expression analyses—across a ­minimum 24-h period. Capturing time-series data with appropriate frequency and duration is essential for robust assessment of circadian-regulated processes, and these designs are commonly referred to as circadian collections. Due to the invasiveness of circadian sampling and biopsies in humans, particularly for those in vulnerable conditions, this work is usually performed using rodent models or biopsy-derived cultured cells *in vitro*. Although circadian collections in musculoskeletal conditions remain limited, for this review, we integrated three publicly available studies encompassing aging [23] and cancer cachexia [24] in mice, and type 2 diabetes (T2D) [25] in human primary cells to assess how disease states impact the expression patterns of the core clock mechanism in skeletal muscle tissue and cells. The general design of these studies included a time-course collection of muscle tissues from mice (aging and cachexia) or from differentiated muscle cells *in vitro* derived from T2D patient biopsies. The sampling frequency and number of biological replicates varied across studies, ranging from every 4 h for 48 h (aging; *n *= 2 per time point per condition), every 4 h for 24 h (cachexia; *n *= 2 per time point per condition), and every 6 h for 42 h (T2D; *n *= 5–7 per time point per condition). While the collections were not identical, the inclusion of multiple time points enabled the extraction of core clock gene expression patterns across a comparable timeline. To facilitate cross-study comparison, we compressed the data into a standardized 24-h window, generating visually comparable references (Figs. 1 and 2; Supplementary File S1).

We began by interrogating the well-defined molecular clock compartment itself. Across aging, cachexia, and T2D, the overall oscillatory expression patterns of the positive limb components *Bmal1/BMAL1* and *Clock/CLOCK* were largely preserved (Fig. 1a–c), suggesting that the core timing machinery, at the mRNA level, was relatively resilient to aging or chronic disease. In contrast, components of the negative feedback arm exhibited greater susceptibility to disruption. In aging mouse muscle, *Per* and *Cry* family members (*Per1*, *Per2*, *Cry1*, and *Cry2*) displayed reduced rhythmic robustness, with attenuated amplitudes and shifts in peak expression timing, despite continued oscillatory behavior (Fig. 1d; Supplementary Fig. S1a and d). These alterations pointed to a weakening of negative feedback precision rather than a complete failure of the molecular timekeeping mechanism.

In cachexia, core clock gene expression patterns were compa­ratively stable, with minimal changes observed in oscillatory profiles, amplitude, or peak timing (Fig. 1b and e; Supplementary Fig. S1b and e). In contrast, primary myotubes from individuals with T2D exhibited a broad dampening of core clock gene amplitudes and altered timing of peak expression, even though only changes in *CLOCK* expression met criteria for statistical loss of rhythmicity (Fig. 1c and f; Supplementary Fig. S1c and f). These findings suggested either amplitude reductions or challenges in maintaining synchronization across the cells, which led to lower and flattened clock gene expression patterns.

Beyond the core clock components, analysis of genes in the auxiliary loop identified that *Rora*, the activator of *Bmal1* expression, was commonly disrupted across all three conditions; the largest disruption was in aging and cachexia, with more modest changes in T2D (Fig. 2a–c). Further analysis of *Nr1d1* and *Nr1d2,* the negative regulators of *Bmal1*, showed disruptions largely in aging and T2D, with minor changes in cachexia (Fig. 2a–c). These observations illustrate that genes involved in circadian clock support are also modified with aging and disease and could impact clock stability and robustness.

**Figure 3:**
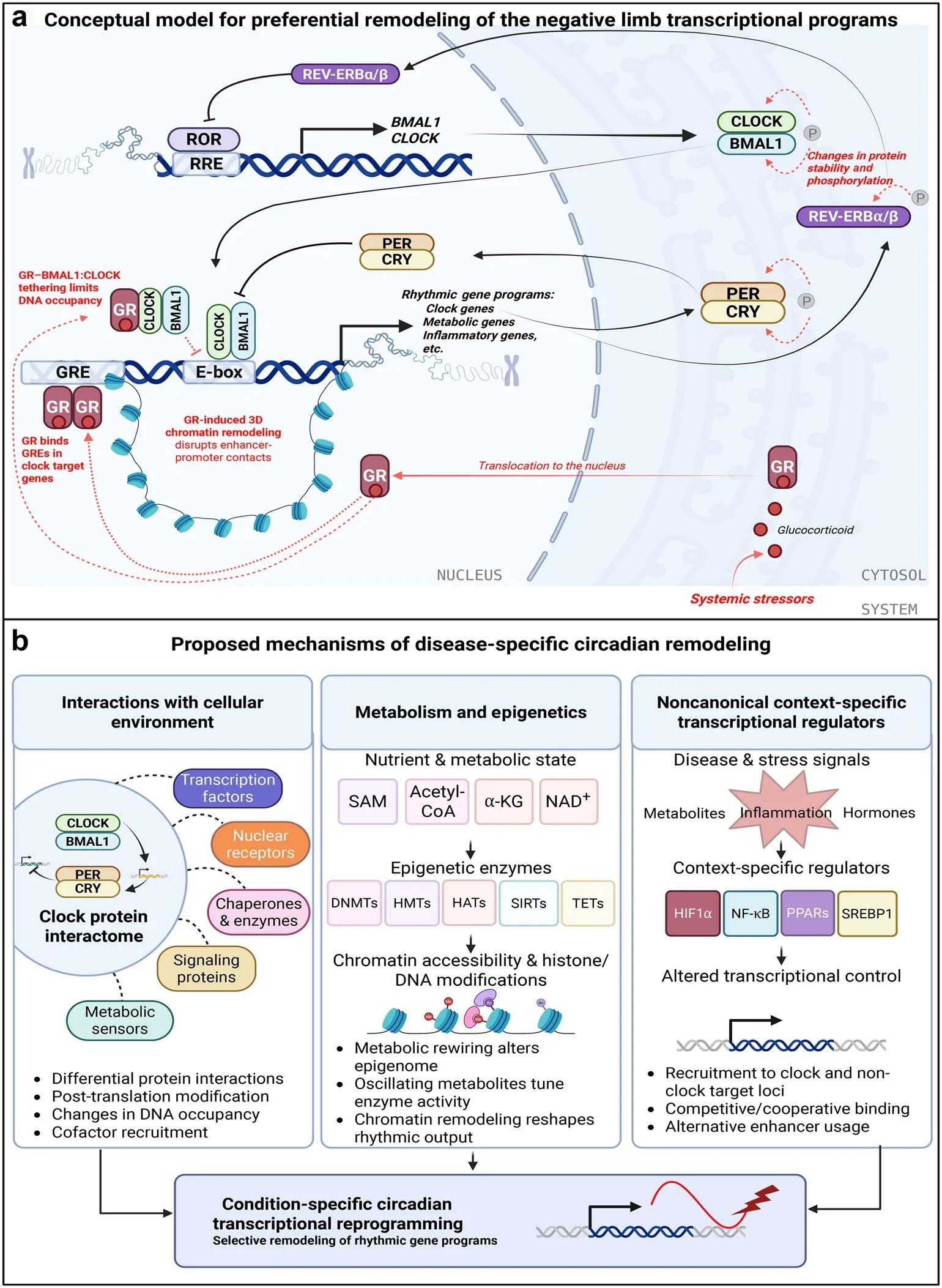
Proposed mechanisms underlying condition-specific remodeling of skeletal muscle circadian transcription. (a) Conceptual model underlying preferential remodeling of the molecular clock negative limb. Under basal conditions, CLOCK:BMAL1 activates rhythmic transcription of core clock genes and downstream rhythmic gene programs through E-box binding, while RORs and REV-ERBα/β regulate *Bmal1* expression through ROR response elements (RREs). We propose that systemic challenges may preferentially remodel the molecular clock negative limb through systemic cues such as GR-dependent mechanisms. Upon glucocorticoid binding, activated GR translocates to the nucleus, where it may (i) bind GREs within clock target genes, including negative limb components; (ii) tether to BMAL1:CLOCK complexes to reduce E-box occupancy; (iii) induce three-dimensional chromatin remo­deling that disrupts enhancer-promoter interactions; and (iv) alter clock protein stability and post-translational modifications. Together, these complementary mechanisms could selectively reshape rhythmic transcriptional programs while preserving much of the core molecular oscillator. (b) Proposed mechanisms contributing to disease-specific circadian transcriptional remodeling. Multiple interacting processes may contribute to the context-specific rewiring of rhythmic gene expression in the skeletal muscle. First, alterations in the cellular environment—including changes in transcription factor availability, nuclear receptor signaling, protein interactions, molecular chaperones, metabolic sensors, and signaling pathways (protein phosphorylation and stability)—may modify clock protein interactions, DNA occupancy, or cofactor recruitment. Second, metabolic remodeling may alter chromatin accessibility through changes in metabolite availability (e.g., S-adenosylmethionine, acetyl-CoA, α-ketoglutarate, and NAD^+^), thereby influencing the activity of DNA and histone-modifying enzymes. Finally, disease- and context-specific transcriptional regulators activated by inflammatory, metabolic, hormonal, or stress-responsive pathways may cooperatively or competitively regulate clock-controlled loci independently of, or together with, the core molecular clock. Collectively, these mechanisms provide a conceptual framework through which distinct pathological conditions may selectively remodel rhythmic transcriptional programs, despite relatively preserved oscillations of core clock genes.

Overall, currently available datasets suggest that the negative and auxiliary limbs of the molecular timekeeping system exhibit more variable expression, suggesting a greater degree of remodeling, while the expression of positive limb core clock factors remains largely intact. Such vulnerability of the negative limb and auxiliary clock components could, in part, reflect their responsiveness to circulating factors, such as cytokines and glucocorticoid signaling. Many components within the negative limb and auxiliary feedback loops contain glucocorticoid response elements (GREs) or cAMP response elements (CREs) in their promoters [26], making their regulation highly responsive to various stimuli. One-way clinical conditions can modulate the expression of the negative limb clock genes via glucocorticoid changes (Fig. 3a). Glucocorticoids are steroid hormones that exhibit robust circadian secretion patterns under healthy physiological conditions, while synthetic glucocorticoids are also widely prescribed as anti-inflammatory therapies, including in the treatment of several cancers [27, 28]. Upon binding to glucocorticoid receptors (GRs) in the cytosol, glucocorticoids trigger receptor translocation to the nucleus, where the receptors bind DNA and promote gene expression by recruiting coactivators. Importantly, alterations in the diurnal regulation of glucocorticoids have been reported in chronic disease states. For example, attenuated diurnal cortisol rhythms with elevated nighttime circulating cortisol levels have been associated with sarcopenia in individuals with T2D [29]. Given their role as potent transcriptional regulators, glucocorticoids represent a plausible mechanism through which systemic physiological stress or pharmacological regimens may preferentially influence circadian clock function via the negative limb. Indeed, acute or chronic alterations in glucocorticoid levels induce phase shifts in peripheral clock gene expression [30, 31] in a dose- and time-dependent manner [32].

In this context, negative limb components—PERs and CRYs—and auxiliary clock components—NR1D1/2 (REV-ERBs) and RORA/B/C (RORs)—may act as the main intermediaries through which systemic cues or stressors reshape rhythmic transcriptional programs without full collapse of the molecular timekeeping system. Indeed, altered sensitivity to and/or levels of glucocorticoids, among other potential mechanisms, are a common feature of many skeletal muscle conditions, including aging [33, 34], cancer cachexia [27, 35], and diabetes [36, 37]. While core oscillator integrity appears preserved, these findings raise the question of how downstream rhythmic programs are affected.

### Aging, cachexia, and T2D drive condition-specific circadian transcriptional rewiring in the skeletal muscle

**Figure 4:**
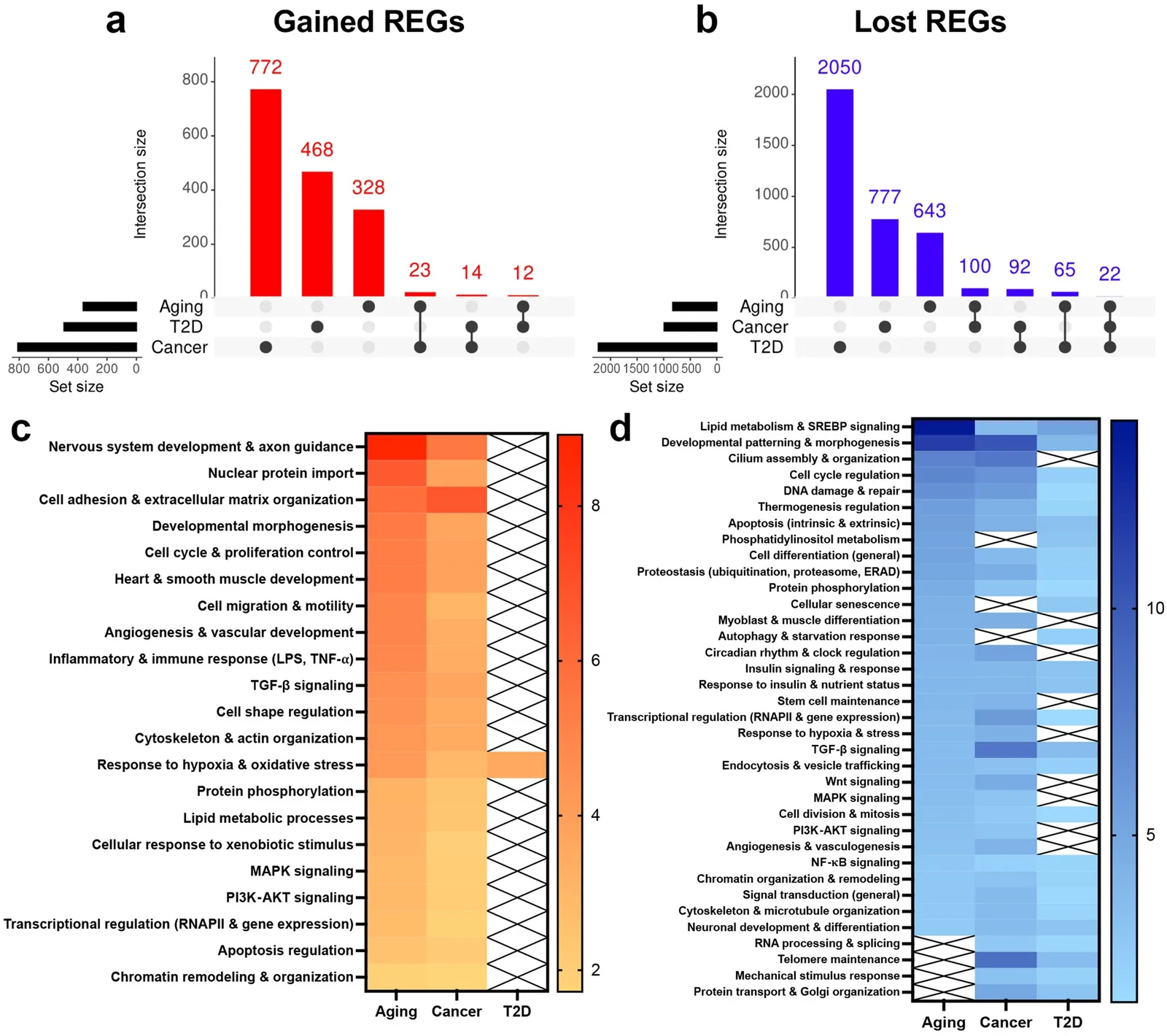
Remodeling of rhythmic gene expression across disease states. (a and b) Summary of gained (a) and lost REGs (b) and their associated biological categories (c and d) across skeletal muscle–affecting conditions, including aging, cancer cachexia, and type 2 diabetes.

Given the essential role of the circadian clock and the redundant regulatory mechanisms that govern cellular timekeeping, the lack of dramatic changes in the molecular timekeeping system of the skeletal muscle exposed to diverse pathological signals is not surprising. In contrast, all three conditions undergo a signifi­cant remodeling of the clock output/rhythmic gene program in muscle tissues and cells. For the integrative analysis of rhythmic gene programs, we relied on the rhythmicity calls provided by each study. Rhythmicity was assessed with cosine-based statistical programs used in the aging and cachexia datasets, and via a non-parametric approach in the T2D dataset, reflecting methodological differences that complicate cross-study comparisons. In all cases, rhythmic genes were defined using study-specific statistical thresholds (e.g., false discovery rate [FDR]-adjusted *P-*values from Cosinor or RAIN). For the aging and cachexia datasets, a raw Cosinor *P-*value < 0.01 was used to determine rhythmicity, while a RAIN FDR < 0.1 was applied in the T2D dataset. Nonetheless, all three datasets revealed substantial aging or disease-specific remodeling in both the number and profiles of rhythmic genes, as well as in the timing of these essential biological processes. A second notable observation is that the genes that gain or lose rhythmicity across these conditions were largely disease-specific (Fig. 4a and b). Across the datasets analyzed here, 49 genes that gained rhythmicity were shared by at least two of the three conditions out of a total of 1617 genes that gained rhythmic expression (Fig. 4a). Similarly, among genes that lost rhythmicity, 279 were shared by at least two conditions of the total 3749 genes that lost rhythmic expression (Fig. 4b). These findings raise the possibility that aging and disease do not simply reprogram circadian output but may destabilize the coherence of rhythmic gene expression, resulting in greater variability and reduced reproducibility of rhythmic transcriptional programs. Future studies are required to define the epigenetic and/or transcriptional mechanisms through which aging or disease modifies the clock output in the muscle and other tissues.

### Condition-specific circadian transcriptional rewiring converges on similar biological processes

Given the limited overlap in rhythmic genes across skeletal muscle subjected to distinct diseases and conditions, a key issue is to elucidate whether these divergent transcripts ultimately regulate shared biological processes or instead drive fundamentally distinct transcriptional programs. To address this, we ran a pathway enrichment analysis using the lists of genes that gained and lost rhythmicity in each condition using DAVID with GO biological process and KEGG pathway annotations. Pathways enriched in each condition were then summarized and clustered into functional categories to avoid duplicated representation, and the highest enrichment score was retained (Supplementary File S2).

Despite this limited overlap at the gene level, the biological processes impacted by these rhythmic changes often overlap substantially (Fig. 4c and d). The overlap in biological processes associated with gained rhythmic genes was most pronounced between muscles of aged and cachectic mice (Fig. 4c). Notably, processes gaining rhythmicity aligned with key hallmarks of aging and cachexia, including extracellular matrix remodeling, inflammatory and immune responses, lipid metabolism, and chromatin organization. This suggests that rhythmic control of these biological pathways may contribute to maladaptive consequences arising from their temporal dysregulation. On the other hand, responses to hypoxia and oxidative stress represented the only biological process shared across all three investigated models (Fig. 4c). The reduced overlap across all three conditions may partly reflect inherent differences between *in vivo* (aging and cachexia) and *in vitro* (T2D) settings. While whole-tissue *in vivo* rhythmic expression analysis reflects the oscillating landscape of the heterogeneous cell populations within the skeletal muscles, *in vitro* experiments using primary myotubes largely represent myogenic cell populations. Additionally, *in vivo* tissue clock systems reflect both intrinsic cell-autonomous clocks as they are influenced by environmental and systemic cues, a layer of regulation that is lost with *in vitro* models.

Biological processes associated with lost rhythmic genes were largely shared among all three muscle-impacting conditions and included lipid metabolism, DNA damage and repair, autophagy, cell cycle regulation, insulin signaling, and transcriptional regulation (Fig. 4d). Supporting the concept of altered rhythmic regulation of autophagy and protein homeostasis, studies using hindlimb suspension—a model of disuse-induced atrophy—and androgen depletion have reported substantial disruptions in the rhythmic expression of autophagy- and atrophy-related mRNAs, accompanied by a loss of coordination among mRNA levels, protein expression abundance, and protein phosphorylation [38–40]. Additionally, disruption of circadian regulation in genes governing mitochondrial turnover (biogenesis and mitophagy), dynamics (fusion and fission), and metabolism has been reported in both T2D primary human myotubes [41] and murine androgen-depletion models [39]. Notably, this includes loss of rhythmic expression of key metabolic regulators, such as NAMPT, the rate-limiting enzyme in the NAD^+^ salvage pathway, and sirtuin 1 (SIRT1), an NAD^+^-dependent regulator of metabolism and circadian function.

Several biological processes are represented in both clusters that gain and lose rhythmic control in all three conditions, including lipid metabolism, cell cycle regulation, and chromatin remodeling. This underscores that temporal regulation of pathways, rather than simple upregulation or downregulation in transcripts, is essential for maintaining homeostasis, while circadian control is a complex process in which both loss and gain of rhythmic gene expression can disrupt temporal control of key pathways for homeostasis. Lipid metabolism exemplifies this vulnerability, as it is tightly governed by multiple layers of circadian control. NR1D1 (REV-ERBα), which shares regulatory targets with BMAL1, functions as a key gatekeeper of lipid metabolism, directly repressing genes involved in lipid synthesis, fatty acid oxidation, and mitochondrial function [42, 43]. REV-ERBα is highly expressed in oxidative muscle fibers, and its loss leads to impaired SIRT1 signaling and reduced mitochondrial biogenesis, ultimately compromising oxidative capacity. In the skeletal muscle, the clock promotes diurnal cycles of lipid storage while coordinately inhibiting lipid and protein catabolism prior to awakening. This occurs via BMAL1-dependent activation of *Dgat2* and REV-ERBα repression of lipid metabolism, linking the role of REV-ERBα in lipid metabolism regulation to its function within the core clock ­machinery [44]. Notably, Sirt1/SIRT1 oscillations appear to be disrupted in all three conditions examined here (Supplementary Fig. S2), further linking circadian dysregulation of transcriptional and metabolic networks to impaired cellular function.

Together, these observations suggest that disease-specific circadian transcriptional rewiring may converge on shared functional modules.

## Differential rhythmicity and temporal coordination of biological processes as an essential component of circadian health

Beyond gain or loss of rhythmic genes, changes in the timing of rhythmic expression represent another layer of circadian dysregulation. While most of the current literature focuses on the presence or absence of rhythmicity in gene expression, circadian regulation goes beyond whether a gene is rhythmic and includes the precise timing of its oscillation. In this context, differential rhythmicity, particularly changes in the phase of peak and trough expression, may have equal or greater physiological relevance than the simple gain or loss of rhythmic expression.

The temporal coordination of molecular processes is regulated to align with predictable physiological demands. For example, in the skeletal muscles from healthy control animals, metabolic pathways and enzymatic activity associated with carbohydrate oxidation and glycolysis often peak around the transition from the rest to the active phase, whereas pathways supporting carbohydrate storage and glycogen synthesis tend to peak later during the active phase [24, 44–47]. This aligns with preparation for the energy demands of the active phase and energy storage following feeding and before the rest phase. Genes involved in lipid meta­bolism have a somewhat similar pattern to those involved in fatty acid transport and some components involved in β-oxidation, with these genes peaking in the mid-late rest phase, whereas genes important for lipid storage peak later in the active phase [44]. These patterns prepare the muscle metabolic profile for the higher energy demands typical of the active phase and temporally assign fuel storage events to the later active phase, following feeding and before rest. In contrast, tumor-bearing mice lose this temporal organization, with diminished rhythmic enrichment of metabolic pathways and the emergence of rhythmic expression in inflammatory and proteasomal processes. These findings suggest that disease states may not only alter which genes are rhythmic, but more importantly disrupt the timing at which key biological processes occur.

Temporal misalignment of biological processes likely has significant consequences for tissue function [48]. Proper circadian regulation ensures that metabolic, repair, and stress-response pathways are activated at appropriate times relative to systemic cues and cellular needs, and are shut down when these processes are not needed. When this timing is disrupted, pathways may be activated or suppressed at the wrong time, thereby impairing metabolic efficiency, exacerbating stress responses, and contri­buting to functional decline, even without complete loss of rhythmic gene expression.

## Mechanisms coordinating disease-specific circadian reprogramming

There are several plausible explanations for why alterations in muscle rhythmic gene programs appear disease-specific. Here, we explore several possible factors that could play a role in this specificity in muscle rhythmic gene program rewiring (Fig. 3b).

### Interactions between core clock factors and the cellular environment

Core clock components dynamically interact with a wide range of proteins—including transcription factors, nuclear receptors, chaperones, enzymes, and transporter proteins [49, 50]—that are themselves tissue- and condition-specific [51]. The stability and likelihood of these interactions may differ across disease states, particularly in conditions that disrupt proteostasis. Changes in protein abundance, post-translational modification status, or subcellular localization could therefore differentially reshape rhythmic gene programs without necessarily altering core clock gene expression. Little to no evidence is currently available to define how unfavorable systemic conditions remodel the interaction dynamics of muscle core clock with other transcriptional regulators. Future studies should therefore move beyond transcript-level analyses and interrogate the clock protein interactomes using protein-protein, protein-DNA/RNA, and/or protein-ligand approaches. Such complementary strategies will be essential to determine whether disease states alter the composition, stability, or genomic context of clock-associated complexes, providing mechanistic insight into how muscle rhythmic genes are rewired in a context-specific manner without noticeable disruption of core clock genes.

### Metabolism and epigenetics

Aging, cachexia, and T2D all share a profound reshaping of tissue metabolism across the system, either directly or indirectly through altered nutrient availability and energy balance.

Genetic models have demonstrated that the skeletal muscle molecular clock through clock output gene regulation plays an active role in regulating metabolic homeostasis. More recently, the intrinsic skeletal muscle clock has been identified as an important metabolic sensor, coordinating nutrient utilization and oxidative metabolism in response to changes in nutrient availability [52]. Consistent with this role, several studies using muscle-specific deletion of *Bmal1* have demonstrated reduced metabolic flexibility through reduced glucose uptake and glucose oxidation [45, 53] while promoting lipid mobilization in the skeletal muscle and reducing diet-induced hepatic lipid steatosis [52]. Mechanistically, regulation of carbohydrate metabolism by the muscle clock appears to be mediated in part through BMAL1:CLOCK regulation of genes such as glucose transporter 4 (*Glut4*) and hexokinase 2 (*HK2*), while interactions between BMAL1 and hypoxia-inducible factor 1 (HIF1) also contribute [54, 55]. BMAL1 directly regulates HIF1α-dependent transcriptional programs involved in glycolysis and oxygen metabolism, providing a molecular link between ­circadian timing, cellular metabolic state, and skeletal muscle fuel utilization [19, 54, 55].

Another mechanism through which metabolic remodeling may, in turn, influence rhythmic gene expression is via alterations in chromatin accessibility and epigenetic regulation. Importantly for this review, we noted that across skeletal ­muscle conditions, biological categories involved in both metabolism and epigenomic regulation were found to gain or lose rhythmi­city (Fig. 4c and d). Because metabolites generated from different metabolic pathways serve as essential cofactors and substrates for the enzymes that write and erase marks on DNA and histones, metabolic change provides a plausible route by which disease states alter the epigenome and potentially the circadian transcriptome [56].

For example, S‑adenosylmethionine (SAM) is the universal methyl donor for DNA methyltransferases (DNMTs) and histone methyltransferases (HMTs), while its product S‑adenosylhomocysteine (SAH) accumulates to inhibit those methyltransferases. Pathological changes and oscillations in one‑carbon nutrients such as folate, methionine, and choline therefore can shift the SAM:SAH balance and overall methylation potential to bias chromatin toward hyper‑ or hypomethylated states [57–59], thereby reshaping epigenetic regulation of gene expression. Additionally, α‑ketoglutarate (α‑KG), a tricarboxylic‑acid (TCA) cycle intermediate, acts as an essential cofactor for various dioxygenase enzymes such as ten-eleven (TET) DNA demethylases and histone demethylases [60]. In contrast, accumulation of TCA‑related metabolites such as succinate, fumarate, or the oncometabolite 2‑hydroxyglutarate (2‑HG) competitively blocks these α‑KG‑dependent enzymes [60–62].

Similarly, acetyl coenzyme A (acetyl‑CoA) serves as the direct acetyl donor for histone acetyltransferases and therefore couples nutrient flux to histone acetylation. One-carbon metabolism (a major supplier of SAM and NADH), as well as NAD and acetyl-CoA pools, is extensively rewired in the context of aging [63–65], cachexia [66], and T2D [67] (reviewed in [68]), providing another potential mechanism by which metabolic dysfunction reshapes the circadian chromatin landscape. Furthermore, NAD/NADH ratio modulates sirtuin deacetylases (SIRTs), linking cellular redox state to the regulation of histone and clock protein acetylation. Together, changes in one‑carbon metabolism, TCA cycle intermediates, acetyl‑CoA availability, and the NAD/NADH ratio can reprogram DNA and histone methylation/acetylation landscapes, thereby perturbing circadian chromatin remodeling and translating metabolic disease signals into altered rhythmic gene expression.

Notably, many epigenetic enzymes themselves exhibit circadian oscillation in healthy conditions, including SIRTs [69], histone acetyltransferases (p300/CBP) [70], and chromatin remodelers (switch/sucrose non-fermentable [SWI/SNF] complexes) [71, 72]. Thus, aging and disease may impact the availability of chromatin-modifying substrates and/or disrupt the rhythmic deployment of the enzymes that write and erase epigenetic marks. Building on this, recent *in vitro* experiments have highlighted that core clock components regulate transcription not only through downstream metabolic cues but also through direct control of chromatin structure. Independently of meta­bolic regulation, the BMAL1:CLOCK complex interfaces with chromatin by recruiting histone acetyltransferases such as CBP/p300 and competing with remodeling complexes such as the BRG1/BRM-associated factor (BAF) complex to modulate nucleosome positioning and accessibility [73, 74]. In line with this, recent studies demonstrate that circadian transcription involves rhythmic nucleosome removal [75], suggesting that temporal control of chromatin architecture is a fundamental mechanism of clock output regulation. This direct regulatory role that BMAL1:CLOCK heterodimer plays at the chromatin level poses an additional mechanistic framework through which circadian timing can be reprogrammed across biological processes, even in the presence of relatively preserved core clock gene oscillations. While direct evidence in the skeletal muscle affecting disease is limited, it is possible that pathological perturbations, such as inflammation, stress signaling, or altered transcription factor availability, could selectively reprogram rhythmic gene profiles by modifying chromatin accessibility, E-box occupancy, or BMAL1:CLOCK cofactor recruitment.

### Alternative mechanisms: evidence from other tissues

Although significant advances have been made in defining circadian regulatory mechanisms in the skeletal muscle, emerging evidence from other tissues suggests that circadian transcriptional programs are more plastic than previously appreciated. Studies across multiple physiological and pathological contexts indicate that rhythmic gene expression can be remodeled without complete disruption of the core molecular clock, raising the possibility that tissue-specific factors and environmental cues may reshape circadian outputs via mechanisms that act through or independently of the canonical clock machinery. Consequently, observations from non-skeletal muscle tissues provide a useful conceptual framework for understanding how circadian transcriptional remodeling may occur in the skeletal muscle during aging, metabolic disease, cancer cachexia, and other conditions.

A recent review examining digestive organ diseases has highlighted the potential role of noncanonical circadian regulators in mediating circadian transcriptional remodeling under stress conditions [18]. In this review, the authors define noncanonical regulators as those that take over circadian transcriptional regulation in the absence of a functional clock or under unfavorable clock conditions, and that can work through both clock-dependent and clock-independent mechanisms. In this framework, noncanonical regulators may influence circadian biology through two non-mutually exclusive mechanisms. First, they may alter the transcriptional activity of the core clock itself by promoting or inhibiting BMAL1:CLOCK chromatin binding through cooperative or competitive transcription factor interactions, chromatin remodeling, or epigenetic regulation. Second, they may directly regulate rhythmic gene expression independent of the canonical clock machinery. Support for the latter mechanism comes from observations that rhythmic transcription can persist, and, in some cases, be extensively remodeled, even when components of the molecular clock are impaired. For example, diet-induced obesity has been associated with extensive circadian enhancer remodeling driven by lipid-responsive transcription factors such as the sterol regulatory element-binding protein 1 (SREBP1) and the peroxisome proliferator-activated receptor α (PPARα) [76], both of which are themselves influenced by circadian regulation.

Among the best-characterized examples of noncanonical regulation of the molecular clock is GR signaling, which provides a mecha­nistic framework for how systemic stress may remodel circadian transcription. Rather than acting solely through classical GREs, GR appears to regulate clock-controlled transcription through multiple complementary mechanisms. GR has been shown to interact directly with the BMAL1 transcriptional complex, limiting its DNA occupancy through tethering-dependent mechanisms and consequently reducing expression of downstream targets such as *Nr1d1* (REV-ERBα). More recently, glucocorticoid signaling has also been implicated in regulating higher-order chromatin architecture at circadian loci. Under stress conditions, GR-dependent chromatin remodeling promotes changes in enhancer–promoter interactions at the *Nr1d1* locus, providing an additional mechanism through which glucocorticoids may reshape rhythmic transcription [77]. Together, these findings suggest that systemic cues may regulate circadian outputs not only by altering transcription factor activity but also by modifying the chromatin environment in which the molecular clock operates (Fig. 3a).

Importantly, GR signaling is unlikely to represent an isolated example. Several nuclear receptors exhibit rhythmic expression, while the secretion of many of their ligands is itself under circadian control. Furthermore, several negative limb and auxi­liary clock components have emerged as important regulators of nuclear receptor function. CRY1/2 interact with approximately one-third of mouse nuclear receptors, with particularly strong interactions reported for PPARs, the vitamin D receptor, and the xenobiotic receptors [78]. Consistent with their canonical role as transcriptional repressors, CRY proteins frequently function as nuclear receptor corepressors [78]. Likewise, PER2 has been shown to coordinate rhythmic transcription through interactions with nuclear receptors including PPARα [79]. Collectively, these observations suggest that nuclear receptors provide an important molecular interface through which hormonal and metabolic signals can remodel circadian transcription.

Beyond nuclear receptor signaling, interactions between noncanonical regulators and core clock proteins have been implicated in circadian remodeling across multiple tissues. For example, coregulation by the inflammasome and REV-ERBα has been linked to circadian remodeling and altered progression of hepatitis. Similarly, in cardiac tissue, formation of a BMAL1-HIF2A transcriptional complex modulates circadian gene regulation and promotes adaptive responses to hypoxic stress, which may be particularly important during recovery following myocardial injury [80]. Additionally, AMPK signaling has been shown to alter the stability of PER and CRY proteins [81] in a tissue- and isoform-dependent manner [82], affecting their activities and, consequently, the expression of clock components and rhythmic genes. Evidence for similar mechanisms is beginning to emerge in the skeletal muscle. The timing of exercise has been shown to remodel rhythmic gene expression, at least in part through induction of HIF1α signaling [83]. Recent feeding-time studies suggest that fat-muscle communication contributes to circadian regulation through adipocyte AMPKα2-dependent signaling pathways [84]. Moreover, inactive-phase time-restricted feeding has also been shown to enhance exercise endurance in mice, in association with diurnal remodeling of gene programs related to mitochondrial oxidative metabolism in the skeletal muscle [85]. Recent work further suggests that clock-associated factors may contribute to context-specific, time-of-day-dependent adaptation to physiological stimuli. For example, REV-ERβ signaling has been implicated in regulating skeletal muscle adaptation to chronic exercise in a time-dependent manner [86], supporting the concept that clock components can shape the transcriptional response to environmental challenges beyond their canonical role in rhythm generation.

Together, these findings suggest that circadian transcriptional outputs arise from dynamic interactions between core clock proteins and tissue-specific regulatory networks. Rather than arising from the molecular clock alone, environmental stimuli, metabolic state, inflammatory signaling, and stress-responsive transcription factors may reshape rhythmic transcriptional outputs by altering chromatin accessibility, transcription factor recruitment, or clock-controlled gene networks (Fig. 3b).

## Challenges and limitations in circadian studies

### Reproducibility of rhythmic gene programs

A key challenge across the circadian field is that rhythmic gene programs, specifically tissue clock outputs, are inherently variable across studies. This issue was recently and elegantly addressed by Brooks *et al.* [87], who integrated 57 publicly available healthy mouse liver time-series RNA sequencing (RNA-seq) datasets and examined both core clock gene expression and rhythmic gene programs. Consistent with our observations, core clock gene expression was highly conserved across all datasets. In contrast, rhythmic gene programs or clock outputs displayed limited robustness in both rhythmic expression and mean phase, with overlap between any two studies never exceeding 60%. Importantly, studies with larger sample sizes showed greater overlap in rhythmic gene profiles, highlighting the importance of adequate replication in time-series experiments to improve reproducibility. Nonetheless, a considerable degree of variabi­lity remained between studies of the liver rhythmic gene transcriptome. The sources of this variation can be linked to possible differences in background strain, age, or sex of the mice, but for many studies it is likely coming from housing and handling conditions including (i) light conditions (i.e., ensuring that mice are on 12-h light/12-h dark prior to collections); (ii) room temperature; (iii) potential factors from different rodent chow; (iv) stressful handling and/or high human traffic in the rooms; and (v) animal facility “cleanliness.” This variability in rhythmic genes among different experiments is a reminder that experiments must include control time-series collections in the same experiment as the control data from other studies will not serve as a robust control.

A similar pattern emerges in the skeletal muscle; however, the number of time-series RNA-seq datasets remains limited. Using control samples from the three muscle-derived datasets examined here, we assessed the reproducibility of rhythmic genes across studies. A total of 941 rhythmic genes were identified in young controls, 1337 in cancer controls, and 2455 in normal glucose tolerance controls from the T2D study (Supplementary Fig. S3). Despite these differences in the total number of rhythmic genes detected, only 485 genes were shared between at least two datasets (Supplementary Fig. S3). This represents approximately 40% of rhythmic genes in young controls, 32% in cancer controls, and 10% in *in vitro* controls with normal glucose tolerance. The lowest overlap between *in vivo* and *in vitro* comparisons is not unexpected, given the species differences, sampling frequency differences, and differences in statistical pipelines. In contrast, the *in vivo* tissue data reflect rhythmic gene expression resulting from both cell-intrinsic and systemic influences, whereas the data from the *in vitro* study largely reflect the cell-intrinsic clock function. While comparing *in vivo* and *in vitro* data demonstrates the variation in both clock gene expression and rhythmic gene output, we note that these experiments have the potential to better define muscle-clock-autonomous regulation (*in vitro*) and how the muscle clock responds to signals from within and outside the tissue. Future studies will require careful design to achieve better alignment but could help identify tissue and systemic targets that can be leveraged for enhancing muscle clock function in health and disease.

## Plastic or maladaptive? How to interpret condition-induced changes in muscle rhythmic gene programs?

Studies examining changes in rhythmic genes within a given tissue across different experimental conditions often rely on seve­ral implicit assumptions in their interpretation, a well-accepted limitation. A canonical assumption is that alterations in rhythmic gene programs observed under disease or aging reflect maladaptive responses that contribute to functional decline. However, relatively little effort has been made to distinguish plastic or adaptive transcriptional changes from those that may actively drive or exacerbate disease, representing a challenge to the field.

Only recently was this assumption directly challenged, albeit not in the context of circadian gene regulation. In a recent preprint, Syed *et al.* [88] proposed that many condition-specific transcriptional changes may represent plastic or adaptive responses to somatic decline, tuning gene expression to maintain organismal stability rather than directly promoting disease phenotypes. While such adaptations may preserve genomic integrity, they can come at a functional cost, including reduced mitochondrial efficiency and metabolic capacity. Indeed, aging and cachexia are characterized by widespread repression of metabolic pathways, often accompanied by declines in proteostasis and mitochondrial function.

If we apply the framework to circadian regulation, we suggest that condition-induced remodeling of rhythmic gene programs in the skeletal muscle may reflect a shift in physiological prio­rities rather than a simple loss of clock function. For example, reduced physical activity and energy demands with aging or disease progression may favor alternative transcriptional programs to support higher-priority processes over metabolic flexibility. In this context, circadian output may transition from a system that anticipates and supports dynamic metabolic demands to one that enforces stability under constrained conditions.

Together, these observations support a model where altered rhythmic gene programs reflect not solely maladaptation, but a context-dependent reorganization of temporal gene expression programs that may be partially protective yet ultimately limit physiological health and performance.

## Closing remarks

**Figure 5:**
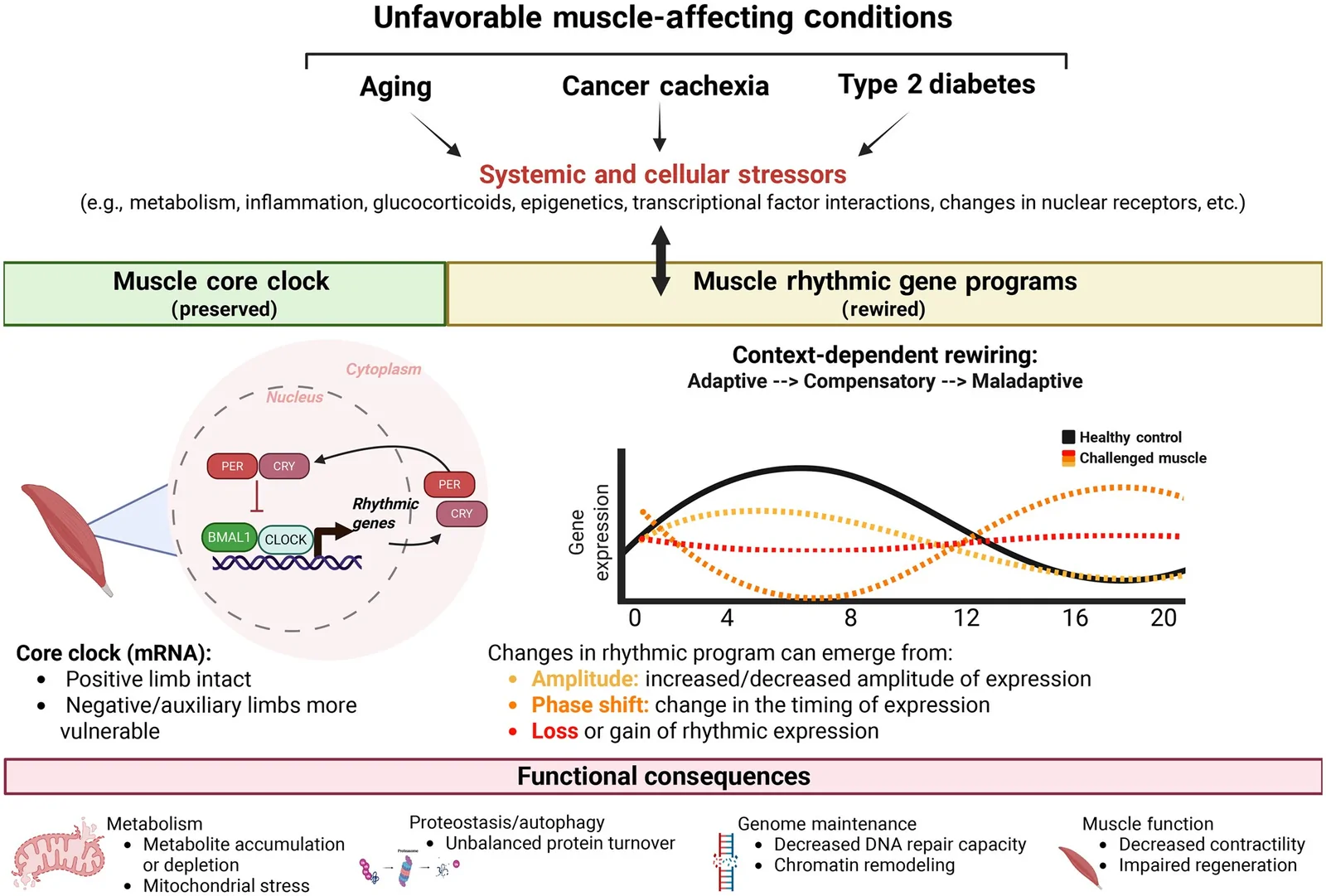
Summary of the proposed model of circadian transcriptional remodeling in the skeletal muscle undergoing unfavorable challenges. Aging, cachexia, and type 2 diabetes are associated with widespread alterations in tissue metabolism, nutrient availability, hormonal signaling, and cellular stress responses. Despite these challenges, expression of the positive limb core molecular clock appears relatively preserved across conditions, whereas rhythmic transcriptional programs undergo substantial remodeling. Changes in rhythmic gene expression may manifest as alterations in rhythm amplitude, phase, or gain or complete loss of rhythmicity, resulting in the rewiring of temporal biological processes. Such remodeling may initially represent adaptive responses that maintain tissue homeostasis under stress; however, persistent or progressive remodeling may become compensatory and ultimately maladaptive. Emerging evidence suggests that systemic cues, including metabolic stress and glucocorticoids, may act through negative limb and auxiliary clock components to reshape circadian outputs without requiring complete disruption of the core molecular oscillator. These changes are proposed to have broad downstream consequences for skeletal muscle physiology, including mitochondrial function, metabolism, epigenetic regulation, and muscle.

Circadian regulation in the skeletal muscle is increasingly recognized as a critical determinant of metabolic health, tissue maintenance, and adaptive capacity. Across aging, cancer cachexia, and T2D, current evidence suggests that circadian disruption does not arise from the collapse of the core molecular oscillator, but rather from instability in the timing, coordination, and deployment of rhythmic gene programs (Fig. 5). This distinction reframes circadian dysfunction as a systems-level problem of temporal organization rather than clock failure. Such instability may reflect both adaptive responses to physiological stress and maladaptive transitions that constrain metabolic flexibility and functional resilience. Moving forward, studies integrating transcriptomic, proteomic, and epigenomic time-series analyses, alongside functional assessments, will be essential to distinguish these possibilities. A clearer understanding of how rhythmic gene programs are rewired in disease may reveal new opportunities to restore temporal coordination and improve skeletal muscle health across diverse pathological conditions.

## Supplementary Material

loag021_Supplementary_Data
