# Supplementary material for: Stable clocks, rewired rhythms: circadian remodeling in skeletal muscle during aging, cachexia, and type 2 diabetes
Source: Life Metab. 2026 Jul 15;5(5):loag021. doi: 10.1093/lifemeta/loag021 (PMC13505632; doi:10.1093/lifemeta/loag021)
Supplement: Supplementary file 1 [file loag021_supplementary_data.zip › Supplemental_Figures_and_legeds - tu.pdf]

Supplementary Figure S1

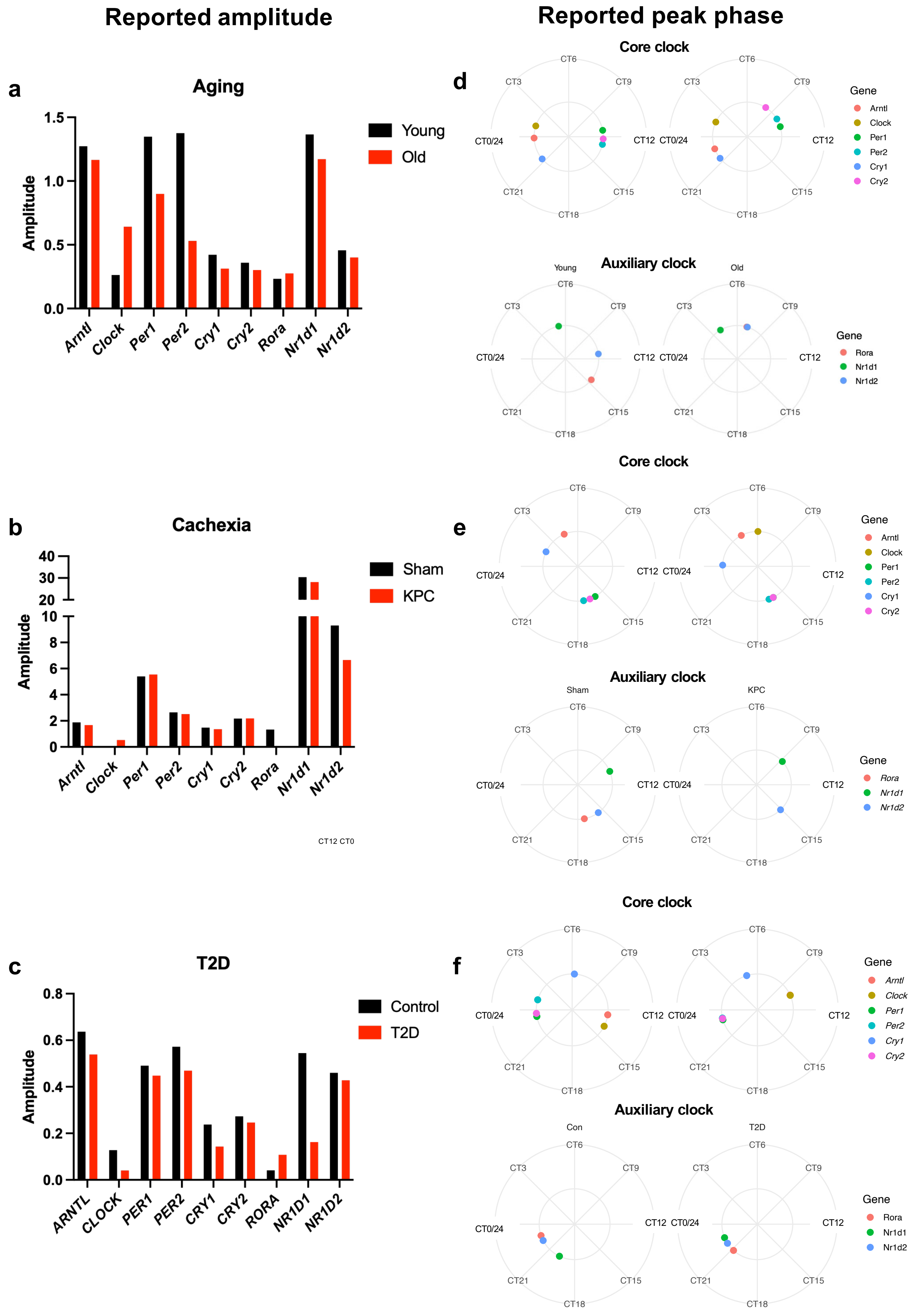

Supplementary Figure S2

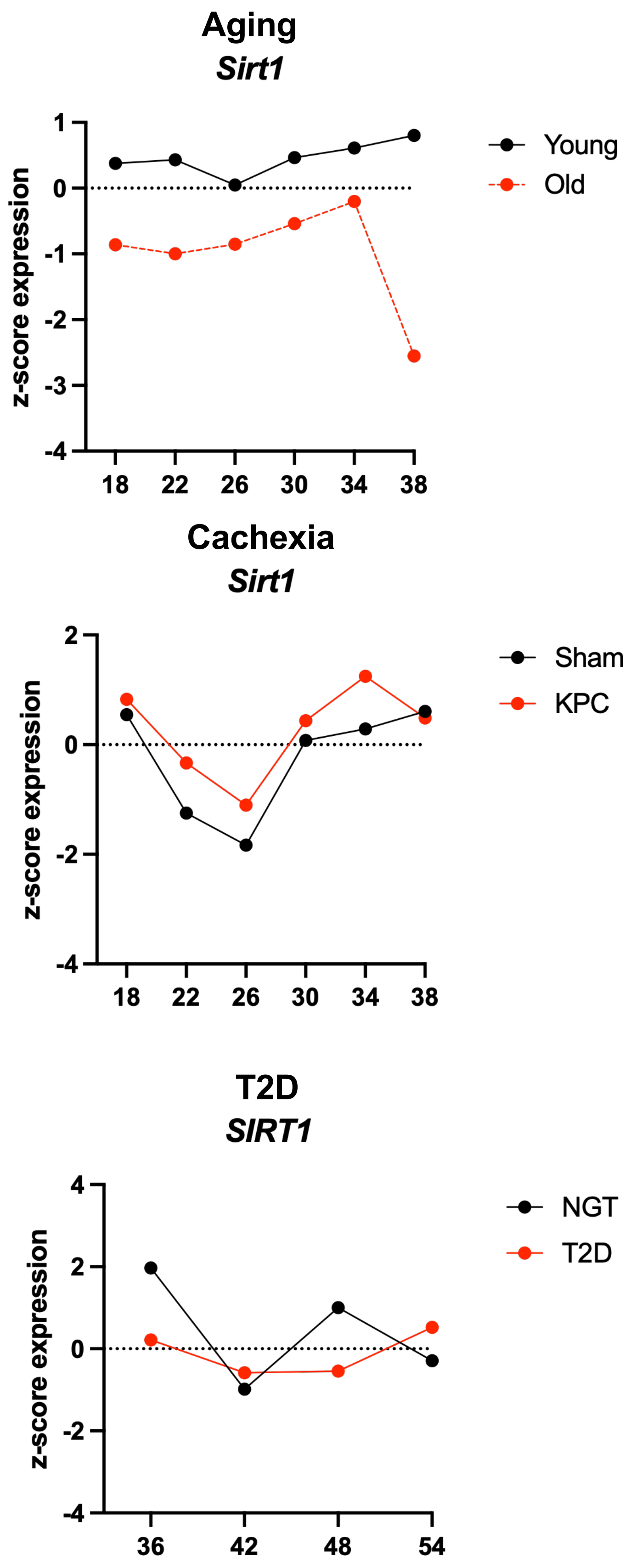

Supplementary Figure S3

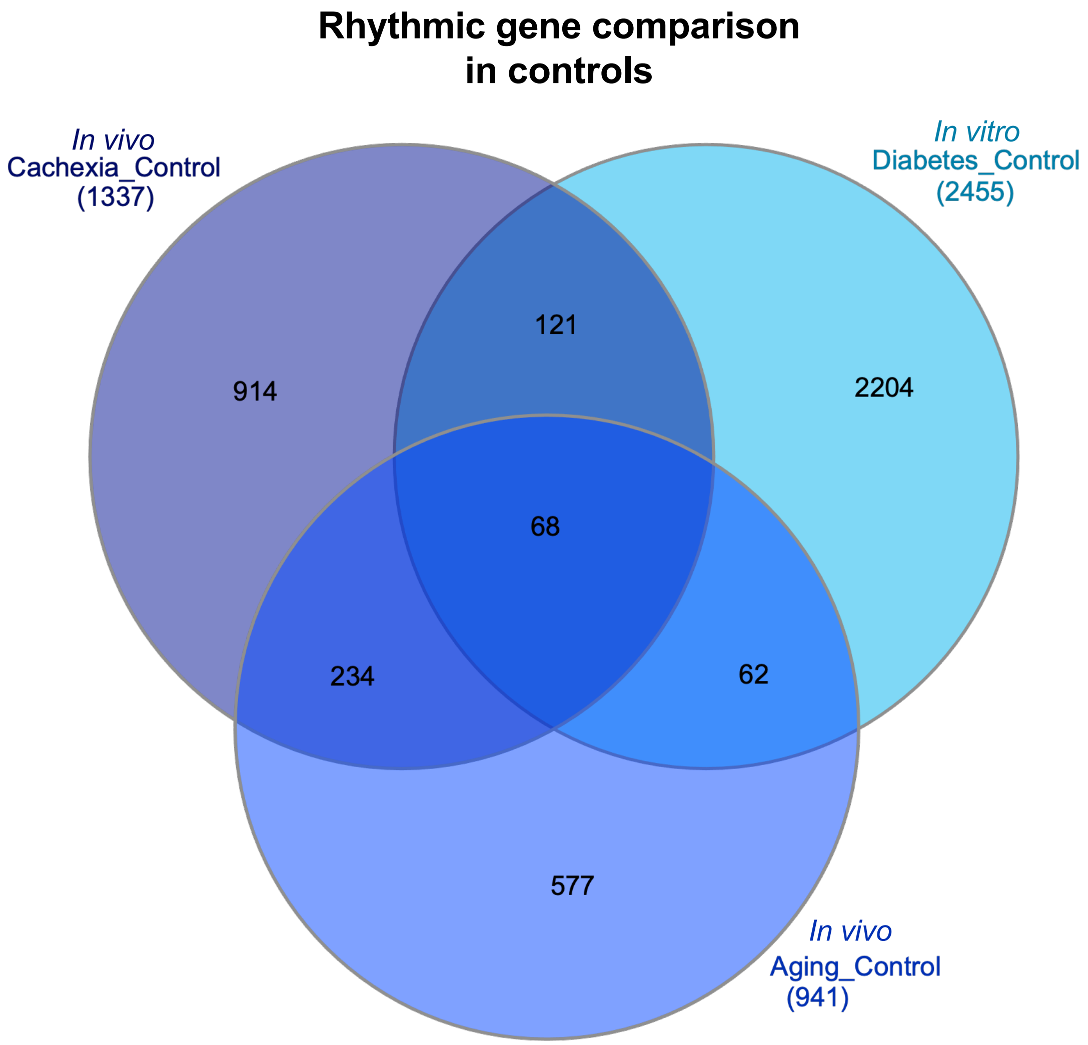

Supplementary Figure S1 *Circadian amplitude and phase of core clock gene expression.*

Amplitude and peak phase of core clock genes derived from published circadian time-course analyses in the skeletal muscle from aging (a and d), cancer cachexia (b and d) , and type 2 diabetes (c and f). Amplitude and peak phase values were extracted directly from the circadian analyses reported in the original publications and were not recalculated from the normalized gene expression profiles shown in Figure 1.

Supplementary Figure S2 *Time of day expression of SIRT1.*

Sirt1/SIRT1 expression in controls and aging muscle, cachectic muscle, and diabetic muscle cells.

Supplementalry Figure S3 *Comparison of rhythmically expressed genes in control muscle tissue/cells.*

Summary of the reported rhythmic genes in the control samples of aging, cancer, and diabetes datasets.

Supplemental File S1 Normalized and z-scored expression profiles of core clock genes, auxiliary clock components, and additional genes featured in this study. This file also includes circadian parameters for clock genes, including amplitude, phase, and peak expression time.

Supplemental File S2 Compiled outputs from rhythmicity analyses reported in the original studies across aging, cancer cachexia, and type 2 diabetes (T2D), along with corresponding functional annotation and enrichment analysis results.
